# Assessment of the Adjuvant Effects of Lentinan on the Tuberculosis Subunit Vaccine BG

**DOI:** 10.3390/vaccines13060597

**Published:** 2025-05-30

**Authors:** Shuai Zhou, Yilin Hou, Xiaojuan Zhang, Zhuoxuan Lv, Quanjie Hu, Xiaobing Yang, Hongxia Niu

**Affiliations:** 1 Key Laboratory of Blood-Stasis-Toxin Syndrome of Zhejiang Province, School of Basic Medical Science, Zhejiang Chinese Medical University, Hangzhou 310053, China; 13075399715@163.com (S.Z.);; 2 Department of Rheumatism and Immunology, Huzhou Third Municipal Hospital, The Affiliated Hospital of Huzhou University, Huzhou 313000, China; 3 School of Basic Medical Science, Lanzhou University, Lanzhou 730000, China

**Keywords:** tuberculosis, subunit vaccine, adjuvant, traditional Chinese medicine adjuvant, lentinan, combined adjuvant

## Abstract

**Objectives**: This study aims to assess the adjuvant effects of lentinan and its combination with Mn(J), a manganese-based colloidal adjuvant, on the BG (fusion protein BfrB-GrpE of *Mycobacterium tuberculosis*) subunit vaccine. **Methods**: A rabbit skin infection model was established to evaluate the immune protection conferred by the BG–lentinan vaccine, the BG–lentinan/Mn(J) vaccine, and the Bacillus Calmette-Guérin (BCG) vaccine against tuberculosis. Rabbits were vaccinated at weeks 0, 2, and 4. Six weeks post-vaccination, antigen-specific IgG levels were measured, followed by a BCG skin challenge. **Results**: Both the BG–lentinan and BG–lentinan/Mn(J) vaccines significantly increased antigen-specific IgG levels against BfrB and GrpE in rabbits (*p* < 0.05). Furthermore, these vaccines accelerated the pathological process following BCG infection. The bacterial load in nodules was notably reduced, with the BG–lentinan vaccine group exhibiting the lowest levels (*p* < 0.01). **Conclusions**: Lentinan and its combined adjuvant, lentinan/Mn(J), significantly enhance the immune response elicited by the BG tuberculosis subunit vaccine, providing effective protection.

## 1. Introduction

Tuberculosis, caused by *Mycobacterium tuberculosis*, is a severe respiratory disease that poses a significant challenge to global public health [1]. The Bacillus Calmette-Guérin (BCG) vaccine is the only widely used vaccine for tuberculosis; however, it offers limited protection in adults [2]. Consequently, the development of new tuberculosis vaccines has become a top priority. Several novel vaccines, including recombinant BCG, live attenuated, viral vector, recombinant protein subunit, and DNA vaccines, are currently in clinical or preclinical development [3]. Among these, protein subunit vaccines are considered highly promising due to their well-defined composition, high safety profile, and ability to enhance the immune protection provided by BCG [4]. Adjuvants are essential components of protein subunit vaccines, as they enhance the immunogenicity of protein antigens, improve protective effects, and reduce the required antigen dosage [5]. Currently, the adjuvants utilized in tuberculosis subunit vaccine research include ASO1E, IC31, GLA-SE, CpG ODN, and BC02 [6]. While these adjuvants have demonstrated progress in clinical trials, they still encounter certain limitations [7]. Therefore, the development of novel adjuvants is critical for advancing the field of tuberculosis subunit vaccine development.

Traditional Chinese medicine adjuvants are considered ideal biological response modifiers because of their diverse biologically active ingredients and broad immune activities [8]. Compared to conventional adjuvants, they have fewer toxic side effects and a lower risk of dependence. As vaccine technologies rapidly advance, researchers are focusing more on developing efficient adjuvant strategies. Given the difficulty of a single adjuvant meeting all ideal immune response criteria, combined adjuvant systems have become a key research focus. These systems combine two or more adjuvants to enhance the immune response through synergistic effects [9]. Various combined adjuvant systems have shown promise in tuberculosis subunit vaccine research, opening new avenues for vaccine adjuvant development [10]. Combined adjuvants are considered optimal for eliciting a more comprehensive immune response. Lentinan (LNT), a (1-3)-β-glucan extracted from the fruiting body of *Lentinus edodes*, exhibits immunomodulatory, anti-tumor, antioxidant, and other beneficial effects [11,12,13]. Studies have shown that lentinan enhances TNF-α and IL-12 production and promotes the generation of Listeria monocytogenes-specific CD8 + T cells [14]. As an adjuvant in the rabies vaccine, lentinan amplifies the immune response, achieving the efficacy of multiple injections with a single dose [15]. Additionally, lentinan has demonstrated potential as an adjuvant in vaccines for Newcastle disease, H5N1, and ovalbumin [15,16,17]. Although lentinan has well-established immunomodulatory properties, its potential as an adjuvant in tuberculosis subunit vaccines remains largely unexplored. Therefore, based on the previously constructed tuberculosis multi-antigen recombinant protein BG [18], this study evaluated the adjuvant effect of lentinan on the tuberculosis subunit vaccine.

Manganese ions (Mn^2+^) have been shown to enhance antigen uptake and presentation by activating the cGAS-STING pathway, thereby promoting both humoral and cellular immune responses with minimal side effects [19]. Their adjuvant potential has been confirmed in vaccines targeting SARS-CoV-2, rabies, and influenza [20,21]. Our previous studies demonstrated that the Mn(J) adjuvant enhances both cellular and humoral immunity induced by BG subunit vaccines, significantly reducing bacterial load and accelerating the healing of immunopathological lesions in vaccinated mice and rabbits [22]. In this study, we further compared the adjuvant effects of lentinan alone and in combination with Mn(J).

## 2. Materials and Methods

### 2.1. Animals

Female New Zealand White rabbits (ordinary grade), weighing 2.0–3.0 kg, were sourced from Yuhang Kelian Rabbit Industry. The rabbits were housed at the Animal Experimental Research Center of Zhejiang University of Traditional Chinese Medicine. During the experiment, the rabbits had ad libitum access to food and water, and bedding materials were changed regularly. The experiment began following a 1-week acclimatization period. The study was approved by the Animal Ethics Committee of Zhejiang University of Chinese Medicine (approval number: IACUC-20231113-04).

### 2.2. Reagent and Instrument

The Bacille Calmette–Guérin (BCG) strain from Denmark was generously provided by the Lanzhou Biological Products Research Institute. Lentinan, a polysaccharide derived from shiitake mushrooms, was obtained from Chengdu Manste Biotechnology Co., Ltd. (Chengdu, Sichuan, China). Mn(J) was supplied by Jiangsu MnStarter Biotechnology Company (Qidong, Jiangsu, China). The goat anti-rabbit IgG antibody (AB6721), conjugated with horseradish peroxidase (HRP), was purchased from Abcam (Cambridge, UK). The acid-fast staining kit was obtained from Shanghai Yuanye Biotechnology Co., Ltd. (Shanghai, China), while pathological staining reagents were provided by the Pathological Laboratory of Zhejiang Chinese Medical University.

### 2.3. Preparation of Fusion Protein BG and Single Antigens BfrB and GrpE

Fusion protein BG and single antigens BfrB and GrpE were prepared as described previously [18]. Briefly, the fusion protein BG and single antigens BfrB and GrpE were overexpressed in *E. coli* BL21 (DE3 pLysS) induced with 0.5 mM IPTG, followed by cell disruption via sonication. Since the fusion protein BG lacks a purification tag, it was purified using ammonium sulfate precipitation and hydrophobic interaction chromatography (HIC). In contrast, His-tagged BfrB and GrpE proteins were purified by Ni-NTA affinity chromatography.

### 2.4. Vaccine Preparation and Immunization Procedures

For the BG–lentinan vaccine, 375 μL of the fusion protein (0.4 mg/mL) was mixed with 750 μL of lentinan (0.1 mg/mL), followed by dilution with PBS to a final volume of 1500 μL. For the candidate vaccine containing lentinan/Mn(J), 375 μL of the fusion protein was mixed with 750 μL of lentinan, followed by the addition of 150 μL of a suspension containing Mn(J). The final volume was adjusted to 1500 μL with PBS. BCG strains were cultured on Lowenstein–Jensen medium. After incubation, the bacterial cells were harvested and adjusted to a concentration of 5 × 10^7^ CFU/mL for subsequent use.

After one week of acclimation, the rabbits were randomly divided into four groups (*n* = 3 per group). Each group was balanced and assigned a distinct immunization method. The first group received a PBS vaccination, the second group was vaccinated with the BCG Denmark strain and served as the control group, the third group received the BG–lentinan vaccine, and the fourth group was immunized with the BG–lentinan/Mn(J) vaccine. The PBS group received an injection of 1000 μL of PBS. The BCG group received 1000 μL (1 × 10^7^ CFU). The BG–lentinan group was vaccinated with 1000 μL containing 100 μg of fusion protein and 500 μg of lentinan. The BG–lentinan/Mn(J) group was inoculated with 1000 μL containing 100 μg of fusion protein, 500 μg of lentinan, and 500 μg of Mn(J). All animals received subcutaneous injections at weeks 0, 2, and 4, except the BCG group, which was injected only once at week 0. The Mn(J) dosage was selected based on our previous studies [22]. The lentinan dosage was informed by studies of other polysaccharide adjuvants, including those from Astragalus and Coriolus versicolor, used in subunit vaccines [23,24].

### 2.5. Detection of Antigen-Specific Antibody Levels in Rabbit Serum

Six weeks after the final immunization, serum samples were collected from rabbits. IgG antibody levels specific to the individual BfrB and GrpE antigens were quantified using ELISA to assess the immune response to each component. Firstly, 96-well plates were coated with 5 μg/mL of BfrB or GrpE and incubated overnight at 4 °C. The plates were then washed with PBS containing 0.05% Tween 20 (PBST) and blocked with 5% BSA at 37 °C for 1 h. Serum samples were initially diluted 1:100, and subsequently serially diluted to a maximum of 1:409,600 or 1:819,200. Anti-BfrB and anti-GrpE antibodies were then added at a dilution of 1:12,000, resulting in a final volume of 100 μL per well. The plates were incubated at 37 °C for 1 h. After washing with PBST, a color developer was added to each well. After a 15-min incubation at room temperature, 100 μL of stop solution was added, and the optical density (OD) was measured at 450 nm.

### 2.6. Rabbit Skin Liquefaction Model and Evaluation of Vaccine Protective Effects

Six weeks after the final vaccination, a 100 μL suspension of *Mycobacterium bovis* containing 5 × 10^6^ CFU was intradermally injected into the subcutaneous tissue on both sides of the rabbits’ backs to replicate the pathological changes seen in the lungs of tuberculosis patients [25,26]. Each rabbit was injected at four points, spaced 2 to 3 cm apart. Pathological changes at the injection sites were observed and recorded daily, including tuberculous granuloma, liquefaction, ulceration, and healing. The criteria for identifying these typical pathological features are as follows: (1) Tuberculous granuloma: The nodules are distinctly red, swollen, and hard in texture. (2) Liquefaction: The nodules begin to soften. Gentle insertion of a sterile syringe needle reveals liquefaction when liquid appears on the needle upon withdrawal, or when gentle pressure is applied, causing the substance to flow out. The peak of liquefaction occurs when the nodule reaches maximum volume, at which point a significant amount of caseous liquid is expelled. (3) Ulceration: The center of the nodule ruptures, releasing a small amount of caseous liquid. (4) Healing: The nodule volume decreases to 60% of its maximum size, and scabbing begins at the rupture site. Complete healing is indicated when the nodule volume reduces to 25% of its maximum size, with visible epithelial formation. Additionally, the width, length, and height of the nodule were measured, and its volume was calculated by multiplying these three dimensions.

### 2.7. Quantification of Bacterial Load in the Skin Nodules

At the peak of liquefaction, a syringe was used to collect as many liquid samples as possible from the lesions. At the end of the healing period, the rabbit was euthanized, and the nodules were excised for homogenization. The collected caseous liquid samples and nodules from the healing period were weighed under sterile conditions, and tissue CFU counts were determined after homogenization. For quantitative CFU analysis, the homogenate was serially diluted and inoculated on Lowenstein–Jensen medium, where it was incubated for three weeks. Finally, the presence of bacteria in the colonies was confirmed by acid-fast staining.

### 2.8. Pathological Examination of Tuberculosis Nodule Sections

Throughout the healing period, nodules and adjacent lesional skin tissue were collected for pathological analysis. Firstly, the dissected nodule tissue was immersed in a 10% tissue fixative solution for 24 to 48 h. After fixation, the tissue was dehydrated with increasing ethanol concentrations, cleared with xylene, paraffin-embedded, and blocked. Next, tissue sections were cut to a thickness of 4–5 microns using an ultra-thin semi-automatic rotary slicer and mounted onto slides. Finally, all slides were stained with hematoxylin and eosin and analyzed using a pathological section scanner.

### 2.9. Statistics and Analysis

Data were analyzed using Microsoft Excel 2019 and GraphPad Prism 8, with the mean ± standard deviation (SD) used to describe the data. A one-way analysis of variance (ANOVA) was performed to compare the mean levels between groups, with Tukey’s post hoc test used for multiple comparisons. Statistical significance was considered at *p* < 0.05.

## 3. Results

### 3.1. Both the BG–Lentinan Vaccine and BG–Lentinan/Mn(J) Vaccine Effectively Induce High Levels of IgG Antibodies in Rabbits

**Figure 1:**
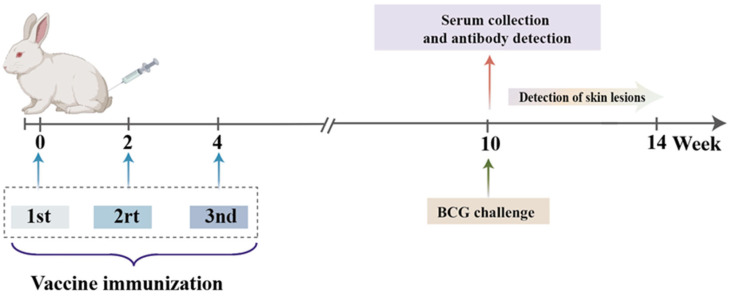
Vaccination procedure for rabbits. The BCG immunization group received a single vaccination at week 0, while the other groups were vaccinated three times at weeks 0, 2, and 4. The antigen-specific immune response was evaluated six weeks after the final immunization. All rabbits received subcutaneous injections.

To evaluate the adjuvant effect of lentinan on the tuberculosis fusion protein BG, we combined fusion protein BG with lentinan or the complex adjuvant lentinan and Mn(J) to prepare the BG–lentinan and BG–lentinan/Mn(J) vaccines. Both vaccines were administered to New Zealand rabbits at weeks 0, 2, and 4 (Figure 1).

**Figure 2:**
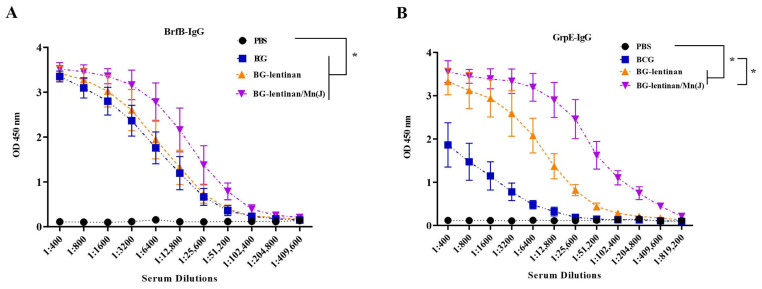
Antigen-specific IgG levels in immunized rabbits. Serum samples were collected six weeks after the final immunization to assess anti-BfrB and anti-GrpE IgG levels. (**A**) BfrB-specific IgG levels. (**B**) GrpE-specific IgG levels. * *p* < 0.05. Data are presented as mean ± standard deviation. Each data point represents an individual animal (*n* = 3 per group).

Six weeks after the final immunization, serum samples were collected from each rabbit (*n* = 3 per group), and IgG antibody levels specific to BfrB and GrpE were measured by ELISA. As shown in Figure 2, rabbits immunized with BG–lentinan or BG–lentinan/Mn(J) vaccines produced significantly higher IgG antibody levels compared to the PBS and BCG control groups, especially for GrpE-specific responses (Figure 2B). Among the subunit vaccine groups, BG–lentinan/Mn(J) induced higher GrpE and BfrB-specific IgG levels than BG–lentinan alone, although the difference was not statistically significant. The higher IgG levels in the BG–lentinan/Mn(J) group suggest that the combination with Mn(J) can enhance the adjuvant effect of lentinan to some extent. These findings demonstrate that both BG–lentinan and BG–lentinan/Mn(J) vaccines effectively elicit strong humoral immune responses, with the combined adjuvant showing a better effect.

### 3.2. Both BG–Lentinan and BG–Lentinan/Mn(J) Vaccines Significantly Shorten the Immunopathological Process Following M. bovis BCG Challenge

**Figure 3:**
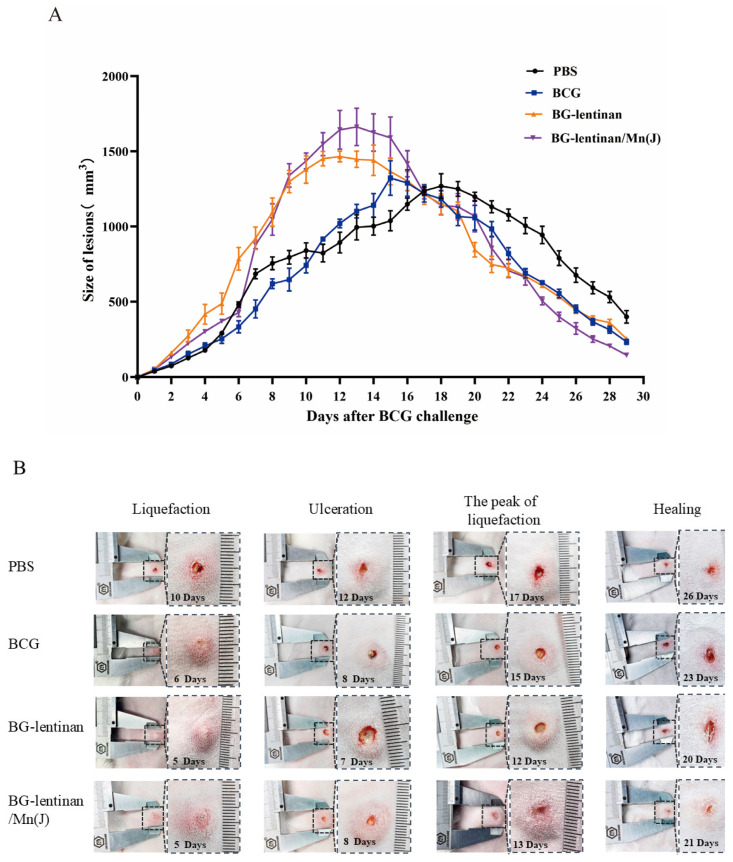
The dynamic progression of skin lesions following *M. bovis* BCG challenge. Six weeks after the final vaccine immunization, 5 × 10^6^ CFU of BCG was injected intradermally at multiple sites on both sides of the rabbit’s skin. The volume of the resulting nodules was measured daily by evaluating their width, length, and thickness. (**A**) Volumes of the skin lesions in different vaccine groups. (**B**) Photographic representation of various pathological stages of tubercles. The figure was generated by the authors. Data represent means of 3 lesions per group.

To further evaluate the protective effect of vaccination, we infected the dorsal skin of rabbits with attenuated *M. bovis BCG* six weeks after the final immunization. This approach simulates *M. tuberculosis* infection in human lungs. To evaluate the impact of the BG–lentinan and BG–lentinan/Mn(J) vaccines on the immunopathological process following tuberculosis infection in the rabbit skin model, we monitored pathological changes at the injection site daily after *M. bovis* BCG infection. We also recorded the volume of skin lesions and the time points at which typical pathological symptoms of tuberculosis appeared (Figure 3, Table 1).

Following the *M. bovis* BCG challenge, the maximum volume of tuberculosis immunopathological lesions varied significantly across different groups (Table 2). Rabbits vaccinated with the BG–lentinan and combined adjuvant vaccines exhibited larger volumes of tuberculosis granulomas. During the peak liquefaction period, the granuloma volumes in the BG–lentinan and BG–lentinan/Mn(J) groups were 1465.43 ± 79.31 mm^3^ and 1662.64 ± 241 mm^3^, respectively, both significantly higher than those in the BCG and PBS groups (Figure 3A). Furthermore, there were significant differences in the onset times of typical pathological symptoms, such as tuberculous granuloma formation, liquefaction, ulceration, and healing. The results showed that these symptoms appeared earlier in the vaccine groups than in the PBS group. Among the vaccine groups, rabbits in the BG–lentinan group showed the earliest liquefaction, ulceration, peak liquefaction, and healing times, occurring on days 5, 7, 12, and 20 post-challenge, respectively. However, the healing process in this group was slower, beginning on day 20 and ending on day 26. In contrast, rabbits vaccinated with BG–lentinan/Mn(J) or BCG exhibited similar liquefaction and ulceration times, occurring on days 5/6 and 8 post-challenge, respectively. Notably, rabbits in the combined adjuvant group healed the earliest, starting on day 21 and completing on day 25, resulting in the shortest total healing duration of 4 days among all groups. Rabbits in the BCG group met the healing criteria on day 23, but full recovery was not achieved until day 28. Overall, both subunit vaccines accelerated the pathological process compared to the PBS group.

### 3.3. Both the BG–Lentinan and BG–Lentinan/Mn(J) Vaccines Significantly Reduce the Bacterial Load at the Tuberculosis Infection Site

**Figure 4:**
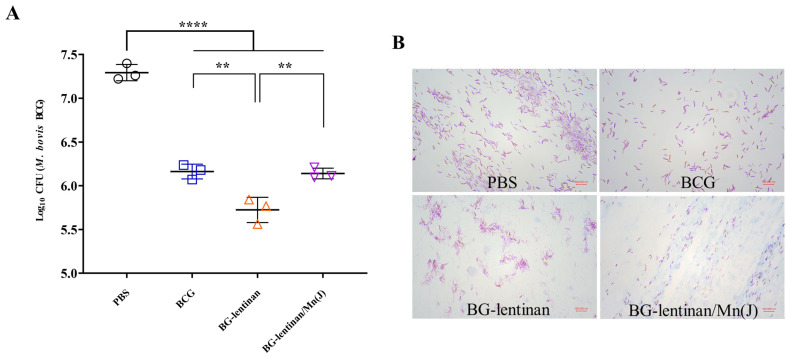
The bacterial load in the caseum necrosis at the peak of liquefaction. Liquefied caseous samples were collected at the peak of liquefaction to assess bacterial load following intradermal BCG challenge. Samples were obtained from rabbits in four groups: PBS (negative control), BCG (positive control), BG–lentinan, and BG–lentinan/Mn(J) vaccine groups. (**A**) The number of tuberculosis bacilli per gram of caseous liquefied sample. (**B**) Acid-fast bacterial staining of cultured caseous liquefied substances. ** *p* < 0.01, **** *p* < 0.0001. Data represent means of 3 lesions per group.

**Figure 5:**
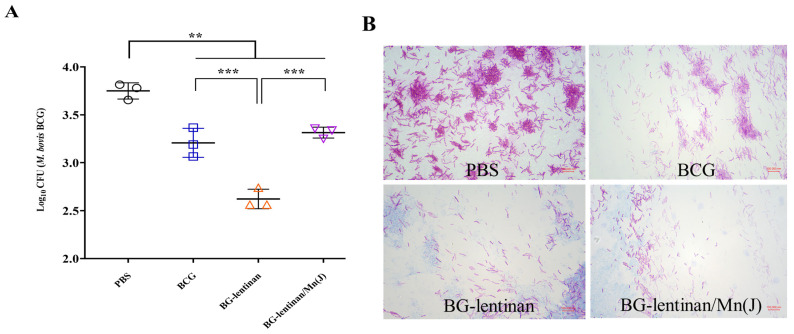
Bacterial load in healing skin nodules. At the complete healing stage, the rabbits were euthanized, and tissue samples from the lesion sites and surrounding skin were collected for hematoxylin and eosin (H&E) staining. Samples were obtained from rabbits in four groups: PBS (negative control), BCG (positive control), BG–lentinan, and BG–lentinan/Mn(J) vaccine groups. (**A**) The number of tuberculosis bacilli per gram of skin nodules. (**B**) Acid-fast bacterial staining of cultured skin nodule homogenates. ** *p* < 0.01, *** *p* < 0.001. Data represent means of 3 lesions per group.

Reducing the bacterial load at infected sites is the most direct indicator of the protective effect provided by vaccines and is considered the gold standard. To evaluate the protective efficacy of the BG–lentinan and BG–lentinan/Mn(J) vaccines, we assessed liquefied samples at the peak of liquefaction and tissue from healing following *M. bovis* BCG infection in the skin (Figure 4 and Figure 5). The results showed that during the peak liquefaction period, the Mycobacterium load in the PBS group was the highest, at 2.0 × 10^7^ CFU/g, while the BG–lentinan vaccine group had the lowest bacterial load, at 5.45 × 10^5^ CFU/g. The BG–lentinan/Mn(J) and BCG groups had bacterial loads of 1.39 × 10^6^ CFU/g and 1.47 × 10^6^ CFU/g, respectively. Notably, the bacterial load in the nodules during the healing period decreased significantly compared to the peak liquefaction period, following a trend similar to that observed during peak liquefaction. Among the groups, the PBS group had the highest bacterial load (5.68 × 10^3^ CFU/g), while the BG–lentinan vaccine group had the lowest (4.27 × 10^2^ CFU/g), followed by the combined adjuvant group (2.07 × 10^3^ CFU/g) and the BCG group (1.68 × 10^3^ CFU/g). To rule out other bacterial contamination, we performed acid-fast staining of liquefied necrotic substances before counting CFU, confirming that all samples contained mycobacteria. A comparison of bacterial loads across groups revealed that both the BG–lentinan and BG–lentinan/Mn(J) vaccines significantly reduced bacterial loads in tuberculous nodules, with the BG–lentinan vaccine showing the most effective protective effect. The protective effect of the BG–lentinan/Mn(J) vaccine was slightly inferior to that of the BG–lentinan vaccine but comparable to that of BCG.

**Figure 6:**
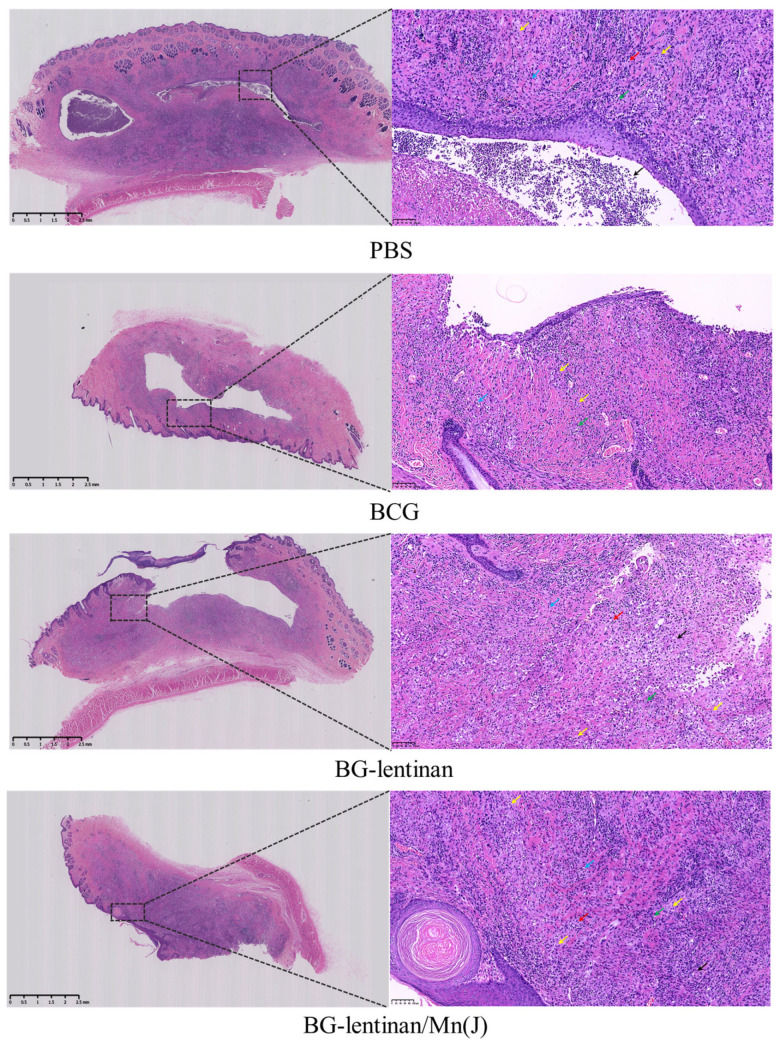
Histopathology of skin lesions after *M. bovis* BCG challenge. The rabbits were euthanized at the end of the complete healing period, and both the lesion site and adjacent skin tissue were collected for HE staining. The results include the original scan at 25× magnification and a localized magnification at 200× of the nodules. Various cells and areas of necrosis in the enlarged pathological sections are indicated with arrows of different colors: red arrows denote eosinophils, green arrows represent lymphocytes, yellow arrows indicate Langhans giant cells, black arrows mark the necrotic areas or abscesses, and blue arrows highlight plasma cells.

Pathological examinations of the skin lesion site were performed during both the peak liquefaction and the complete healing period. Hematoxylin–eosin (HE) staining revealed that pathological changes in skin lesions across all groups were consistent with mycobacterial infection, characterized by inflammatory cells, lymphocyte infiltration, liquefied necrotic tissue, and Langhans giant cells (Figure 6). These findings further substantiate the efficacy of the tuberculosis replacement rabbit model. Notably, no significant differences in pathological changes were observed among the groups during the healing period.

## 4. Discussion

Traditional Chinese medicine adjuvants, such as polysaccharides, saponins, and flavonoids, are known for their high safety and low dependency. These adjuvants effectively reduce the required antigen dose, extend immune protection duration, and significantly enhance both cellular and humoral immune responses, highlighting their substantial developmental potential [7,27]. This study evaluated the adjuvant effect of lentinan, both alone and in combination with Mn(J), in a tuberculosis subunit vaccine. The results showed that both adjuvants significantly enhanced the antibody response of rabbits to BfrB and GrpE antigens, while also accelerating the pathological process after infection with the attenuated *M. bovis BCG* strain in the skin, reducing bacterial load in the nodules. As a result, lentinan show promising potential for use in the development of tuberculosis vaccines.

Selecting an appropriate animal model is crucial for the development of tuberculosis vaccines [28]. While mice, guinea pigs, and primates offer distinct research advantages, they have limitations in accurately simulating the pathology of human tuberculosis [29,30,31]. In contrast, rabbits exhibit delayed hypersensitivity reactions, liquefaction, cavity formation, and tuberculous granulomas following *M. tuberculosis* infection, closely resembling human tuberculosis manifestations and facilitating experimental manipulation [32]. However, the need for Animal Biosafety Level 3 conditions for tuberculosis infection, coupled with the larger size of rabbits, limits the broader application of this model in vaccine research. Our previous study found no significant difference in the liquefaction of skin tuberculosis caused by infection with attenuated *M. bovis BCG* compared to that induced by virulent strains [33]. Building on this foundation, the present study assessed the effects of BG–lentinan and BG–lentinan/Mn(J) vaccines in a rabbit skin BCG infection model.

This study compares the immunogenicity and protective efficacy of the BG–lentinan and BG–lentinan/Mn(J) vaccines in rabbits, highlighting the potential of both single and complex adjuvants in tuberculosis vaccine development. The results show that both the BG–lentinan and BG–lentinan/Mn(J) vaccines significantly enhance IgG antibody levels against BfrB and GrpE antigens in rabbit serum, emphasizing the immune-boosting effects of traditional Chinese medicine adjuvants. Notably, the BG–lentinan/Mn(J) vaccine exhibits a greater capacity to elevate GrpE-specific IgG antibody levels. In terms of protective efficacy, the BG–lentinan group showed the lowest bacterial load during both the peak and healing period of lesion liquefaction, indicating a strong anti-tuberculosis response. In contrast, the BG–lentinan/Mn(J) group exhibited a faster healing rate. Moreover, no additional bacterial contamination was detected through acid-fast staining and microscopy, confirming that the bacteria in the lesion liquefaction were mycobacteria. Histopathological examinations showed that the pathological changes in skin lesions across all groups were consistent with mycobacterial infection, supporting the use of rabbits as a suitable alternative animal model for tuberculosis research.

Although *M. tuberculosis* primarily combats infection through T-cell-mediated immunity [34], studies have shown that antibody production is equally crucial in preventing tuberculosis [35]. Pre-treatment with specific antibodies or vaccination can significantly reduce the *M. tuberculosis* load and prolong the survival of infected animals [36,37]. Other studies have shown that vaccine-induced antibody levels are strongly correlated with the reduction of *M. tuberculosis* [38,39]. This study further confirms that lentinan, especially when combined with Mn(J), significantly enhances the production of antigen-specific antibodies, reduces bacterial load, and accelerates healing at infected sites. These findings substantiate the correlation between vaccine-induced specific antibodies and their protective effects, providing a solid foundation for the design and improvement of tuberculosis vaccines.

Our evaluation of protective efficacy showed that all subunit vaccinations promote liquefaction and healing at the lesion site, while also reducing bacterial load in liquefaction pathology. This effect may be attributed to the vaccine’s ability to trigger a robust immune response at the infection site, similar to the mechanism described in the Koch phenomenon. Notably, the BG–lentinan/Mn(J) vaccine excels at enhancing IgG antibody levels and accelerating healing, suggesting that the combined adjuvant may further amplify the immune response through synergistic effects. However, the specific mechanisms through which different adjuvant components contribute to the immune response require further investigation. We speculate that the combination of the two adjuvant components may activate additional signaling pathways and cellular responses, leading to a stronger immune response compared to lentinan used alone.

Lentinan, a fungal-derived β-glucan, has shown strong immunoenhancing effects, good safety, and low dependency in vaccine studies involving rabies, Newcastle disease, H5N1 influenza, and ovalbumin. In this study, we found that lentinan exhibits a certain adjuvant effect in tuberculosis subunit vaccines. Mechanistically, it might bind to Dectin-1 receptors on dendritic cells (DCs) and macrophages [40], activating the Syk-Card9-Bcl10-MALT1 axis, which triggers NF-κB and MAPK (p38, ERK, JNK) pathways [41]. This promotes DC maturation, upregulates co-stimulatory molecules (CD80, CD86), enhances antigen presentation, and drives CD4^+^ T cell differentiation, particularly toward Th1 and Th17 subsets [42]. Lentinan also stimulates macrophage phagocytosis, antigen processing, and secretion of cytokines such as IL-12, TNF-α, and IL-6, thereby linking innate and adaptive immunity [43]. Beyond enhancing primary immune responses, lentinan may also support the induction and maintenance of immunological memory. Lentinan has been shown to promote T follicular helper (Tfh) cell differentiation, facilitating germinal center formation and the generation of memory B cells and long-lived plasma cells [44,45,46]. Moreover, lentinan-based adjuvants such as GO-LNT/OVA have been shown to prolong antigen presentation and immune activation relative to GO/OVA, thereby supporting sustained humoral responses [47]. Due to the limited availability of rabbit-specific reagents, immune memory responses were not assessed in this study. Future research will focus on assessing the role of lentinan in the induction, maintenance, and functional activation of memory responses to better clarify its potential as a long-term immunostimulatory adjuvant for tuberculosis subunit vaccines.

In summary, this study assessed the adjuvant effect of lentinan and its combination with Mn(J) in a tuberculosis subunit vaccine using a rabbit model. The findings indicate that these two adjuvants significantly enhanced the immune response in rabbits against BfrB and GrpE. The specific immune response generated effectively reduces bacterial load following mycobacterial infection and accelerates the healing process at the infected site. Furthermore, as a vaccine adjuvant, lentinan has demonstrated high efficiency, safety, and low dependency in vaccines for rabies, Newcastle disease in chickens, H5N1 influenza, and ovalbumin [9,15,16,17]. This suggests that lentinan has the potential to serve as an adjuvant in tuberculosis vaccines. Certainly, in subsequent studies, it is necessary to use virulent tuberculosis strains to further verify the adjuvant effect of lentinan and to determine its optimal dosage.

## Figures and Tables

**Table 1:** Immunization groups and vaccination protocols.

	Injection Route	Dose	Number of Immunizations	Challenge Time	Challenge Route	Challenge Site	Challenge Dose
PBS	Subcutaneous injection	1000 μL of PBS	3	6 weeks post-immunization	Intradermal injection	Dorsal skin	100 μL suspension of BCG containing 5 × 10^6^ CFU
BCG	Subcutaneous injection	1000 μL of BCG (1 × 10^7^ CFU)	1	6 weeks post-immunization	Intradermal injection	Dorsal skin	100 μL suspension of BCG containing 5 × 10^6^ CFU
BG–lentinan	Subcutaneous injection	1000 μL (100 μg of fusion protein and 500 μg of lentinan)	3	6 weeks post-immunization	Intradermal injection	Dorsal skin	100 μL suspension of BCG containing 5 × 10^6^ CFU
BG–lentinan/Mn(J)	Subcutaneous injection	1000 μL (100 μg of fusion protein, 500 μg of lentinan, and 500 μg of Mn(J))	3	6 weeks post-immunization	Intradermal injection	Dorsal skin	100 μL suspension of BCG containing 5 × 10^6^ CFU

**Table 2:** Effects of different vaccine candidates on *M. bovis* BCG-induced rabbit skin lesions.

	Maximal Granuloma Size (mm³) (Means ± SD)	Initiation of Liquefaction (Days Post-Infection)	Onset of Ulceration (Days Post-Infection)	Time when Liquefied Area was Maximum (Days Post-Infection)	Lesion Volume (mm³) when the Liquefied Area was Maximum (Means ± SD)	Healing (Days Post-Infection)	Complete Healing (Days Post-Infection)
PBS	794.19 ± 90.7	10	12	17	1268.29 ± 82.59	26	29
BCG	252.96 ± 81.3	6	8	15	1322.47 ± 302.4	23	28
BG–lentinan	415.69 ± 131.5	5	7	12	1465.43 ± 79.31	20	26
BG–lentinan/Mn(J)	301.64 ± 26.2	5	8	13	1662.64 ± 241	21	25

## Data Availability

All data generated or analyzed during this study are included in the published article. Additional datasets used and/or analyzed during the current study are available from the corresponding author upon reasonable request.
